# Non-Antibiotic Drug Repositioning as an Alternative Antimicrobial Approach

**DOI:** 10.3390/antibiotics11060816

**Published:** 2022-06-17

**Authors:** Alexia Barbarossa, Antonio Rosato, Filomena Corbo, Maria Lisa Clodoveo, Giuseppe Fracchiolla, Antonio Carrieri, Alessia Carocci

**Affiliations:** 1 Department of Pharmacy-Drug Sciences, University of Bari “Aldo Moro”, 70125 Bari, Italy; alexia.barbarossa@uniba.it (A.B.); antonio.rosato@uniba.it (A.R.); filomena.corbo@uniba.it (F.C.); giuseppe.fracchiolla@uniba.it (G.F.); alessia.carocci@uniba.it (A.C.); 2 Interdisciplinary Department of Medicine, School of Medicine, University of Bari “Aldo Moro”, 70124 Bari, Italy; marialisa.clodoveo@uniba.it

**Keywords:** antibacterial activity, antifungal activity, non-antibiotic agents, antimicrobial resistance, repositioning

## Abstract

The worldwide scenario of antibiotic resistance and the falling number of funds for the development of novel antibiotics have led research efforts toward the study of specific cost-effective strategies aimed at discovering drugs against microbial infections. Among the potential options, drug repositioning, which has already exhibited satisfactory results in other medical fields, came out as the most promising. It consists of finding new uses for previously approved medicines and, over the years, many “repurposed drugs” displayed some encouraging in vitro and in vivo results beyond their initial application. The principal theoretical justification for reusing already existing drugs is that they have known mechanisms of action and manageable side effects. Reuse of old drugs is now considered an interesting approach to overcome the drawbacks of conventional antibiotics. The purpose of this review is to offer the reader a panoramic view of the updated studies concerning the repositioning process of different classes of non-antibiotic drugs in the antimicrobial field. Several research works reported the ability of some non-steroidal anti-inflammatory drugs (NSAIDs), antidepressants, antipsychotics, and statins to counteract the growth of harmful microorganisms, demonstrating an interesting winning mode to fight infectious diseases caused by antimicrobial resistant bacteria.

## 1. Introduction

The research and the development of new drugs is currently an onerous, laborious, and uncertain process, as can be deduced from the restricted number of novel drugs approved every year. Indeed, even those molecules that advance in clinical trials can often fail in the final stages of testing. An excellent strategy to overcome these problems, diminish failure rates and the related costs is drug repositioning or repurposing. These two expressions refer to the approach of delving into existing drugs for use in new therapeutic indications. The success rate of drugs repurposing method accounts for almost 30% of new FDA-approved drugs in recent years [1]. Several procedures can be exploited for the identification of the best candidates for drug repositioning, including new screening platforms and advanced in silico and bioinformatic studies [2]. Countless stories of drug repositioning are well reported in the recent literature, such as those of finasteride, thalidomide, sildenafil, metformin, hydroxychloroquine [3,4,5]. For instance, one of the main applications of drug repurposing in recent times has been the COVID-19 pandemic. Indeed, several existing molecules, such as remdesivir, ivermectin, lopinavir/ritonavir, baricitinib, dexamethasone, have been evaluated and used for their therapeutic potential against coronaviruses [6,7,8]. In addition, many recent reports have documented an increase in the number of multi-resilient organizations (MDGs) during the COVID-19 pandemic. The MDROs include carbapenemase-producing Enterobacterales, *P. aeruginosa*, multidrug-resistant *Acinetobacter baumannii*, methicillin-resistant *Staphylococcus aureus* (MRSA), pan-echinocandin-resistant *Candida glabrata,* and multi-triazole-resistant *Aspergillus fumigatus*. The reason is multifactorial and could be attributed, in particular, to high rates of antimicrobial agent utilization in COVID-19 patients, with a relatively low rate of co- or secondary infection [9,10,11]. Moreover, in the last few years, hundreds of pathogens, such as bacteria, fungi, and protozoa, have emerged or re-emerged as a cause of intractable infectious diseases. Although several research efforts are trying to solve this issue, the obstacles mentioned above, primarily the excessive cost connected with the development of a new drug, make it difficult to discover and market new molecules. Moreover, some infectious diseases, for which a therapy already existed (such as tuberculosis, pneumonia, and malaria), have lost responsiveness to the treatment, leading to the phenomenon of antimicrobial resistance [12]. The opportunity of drug repurposing or drug combinations seems to be a potential solution to combat resistance development in serious infectious diseases [13,14,15,16]. In particular, therapeutic agents not originally designed for antibiotic or chemotherapeutic purposes but that subsequently demonstrated antimicrobial properties are classified under the name of “non-antibiotics” [17]. Among them, antihistamines [18,19,20], tranquilizers [21], antihypertensives [22], antipsychotics [23,24,25,26], and anti-inflammatory agents [27,28] represent classes of drugs that unexpectedly showed antimicrobial effects. In particular, serotonin reuptake inhibitors have been found to be effective. Among these drugs, the one that stands out is sertraline, possessing antimicrobial potentials, which is able to enhance the activity of several antibiotics, reverse multidrug-resistant phenotypes of bacteria, and make them susceptible to previously resistant drugs. Table 1, Table 2, Table 3 and Table 4 present a summary of the different classes of non-antibiotic drugs that have shown antimicrobial activity documented in scientific papers recently published and reported in this review.

## 2. Non-Steroidal Anti-Inflammatory Drugs (NSAIDs) as Antimicrobial Agents

A number of non-antibiotic drugs have been tested and shown to have some influence on the physiology and viability of microorganisms. Included in these drugs are non-steroidal anti-inflammatory drugs (NSAIDs) (Table 1), which represent the best known and most widely used class of drugs in the world. Acetaminophen (belonging to NSAIDs class but with a weak anti-inflammatory capacity), acetylsalicylic acid, and other NSAIDs, such as diclofenac and ibuprofen, are some of the most used drugs [29]. In addition to their anti-inflammatory action, they have analgesic and antipyretic properties [30]. It has also been shown but neglected for over 20 years that NSAIDs do have direct and indirect antimicrobial effects [16,31,32]. The primary mechanism deemed responsible for the antimicrobial activity of NSAIDs refers to their behavior as protonophores [33].

The infections caused by *Candida* spp. fungal pathogens are the principal agents of several biomaterial-related infections, especially related to biofilm formation [34]. In 2016, Rosato et al. reported in their study on the association between echinocandin anidulafungin (ANF) and some NSAIDs against nine *Candida* strain biofilms: four *Candida albicans*, two *Candida glabrata,* and three *Candida guilliermondii*. Their results outlined that ANF was effective alone against biofilm cells with a minimum inhibitory concentration (MIC) value amounting to 2 µg/mL. Furthermore, they proved an interesting synergistic effect between ANF and three NSAIDs: aspirin, diclofenac, ibuprofen. Indeed, the fractional inhibitory concentration index (FICI) values resulted in values of less than 0.5. The three NSAIDs were also tested alone. The inhibition of biofilm growth by aspirin was more evident at concentrations ranging between 0.2 mM and 1 mM. On the contrary, Ibuprofen possessed a lower effect than aspirin against *C. albicans* strain biofilms, whereas its activity against *C. glabrata* and *C. guilliermondii* was noticeable. Diclofenac inhibited the tested biofilms to a lesser level, at 100 mM [35]. More recently, the same research group combined the antimicrobial effect of *Mentha piperita*, *Pelargonium graveolens*, and *Melaleuca alternifolia* essential oils (whose antimicrobial activity has been extensively reported in the literature) with that deriving from diclofenac sodium salt (DSS). When tested alone, DSS achieved MIC values ranging from 1.02 to 2.05 µg/mL. Conversely, this value was significantly reduced through the association between DSS and *Pelargonium graveolens* (with FICI values from 0.23 to 0.35, demonstrating a synergistic effect) or *Mentha piperita* (FICI = 0.22–0.30) essential oils, resulting in a MIC value amounting to 0.05 µg/mL. The most susceptible of the strains tested was found to be *C. parapsilosis*, both from ATCC and clinical isolation [36]. Another recent study focused the attention on the antibiofilm effect of diclofenac and antibiotic solutions in endodontic therapy. In particular, the purpose was to make a comparison between the antibiofilm activities of a triple antibiotic solution (or TAS, composed of minocycline, metronidazole, and ciprofloxacin), a double antibiotic solution (or DAS, containing metronidazole and ciprofloxacin), and diclofenac solutions at different percentages against *Enterococccus faecalis* biofilm, using eighty-four sterile radicular dentin blocks as substrate. Tests were carried out for a contact time of 5 min. The reduction percentage of the colony-forming unit (CFU) was in a range of 62.98 and 98.62, respectively, for TAS and diclofenac solutions. The latter demonstrated a concentration-dependent activity. The research group also performed experiments by using the confocal laser scanning microscopy, in which the log10 total biovolume in all groups resulted in very similar values and exhibited a poor but important decrease with respect to the control; 5% and 2.5% DCSs gave the lowest viable cell percentage. The TAS and DAS groups displayed intermediate values without considerable differences [37].

Ibuprofen, another NSAID, hampers the growth of *Escherichia coli* at therapeutic levels and, at low pH, also of *S. aureus*, *Microsporum* spp., and *Trichophyton* spp. [16,38]. Furthermore, in 2018, Shah et al. proved the antimicrobial activity of ibuprofen against *Pseudomonas aeruginosa* and *Burkholderia* spp. strains, assessing the dose-dependent activity by measuring the endpoint number of colony-forming units (CFUs) and growth kinetics. Moreover, in an in vitro *P. aeruginosa* biofilm model, ibuprofen decreased the rate of biomass accumulation over 8 h of growth. Instead, the oral delivery of ibuprofen has been evaluated in an acute *Pseudomonas* pneumonia model. After intranasal inoculation, ibuprofen-treated mice showed decreased CFU counts and enhanced survival with respect to the control animals. Preliminary biodistribution studies after ibuprofen aerosol delivery demonstrated a fast accumulation of ibuprofen in serum and minimum retention in lung tissue and bronchoalveolar lavage fluid. The research group prepared ibuprofen-encapsulated polymeric nanoparticles (Ibu-NPs) to ameliorate the pharmacokinetic profile, resulting in the inhibition of growth of *P. aeruginosa* in vitro [33]. In 2020, Pereira et al. prepared and evaluated epichlorohydrin-crosslinked chitosan microspheres for Ag^+^ adsorption and formation of silver nanoparticles (AgNPs). This system was used to incorporate ibuprofen and demonstrated an interesting activity against *E. coli* and *S. aureus*. Furthermore, the microspheres with AgNPs released more drug (77%) than the material without AgNPs [39]. Another study demonstrated the capability of some cyclooxygenase inhibitors (ibuprofen and celecoxib) to affect *Mycobacterium tuberculosis* infection in aerosol-challenged mice. The authors found that this activity could lead to impairments of the Type-1 helper (Th1) T-cell response, as CD4 T cells in COXi-treated animals significantly decreased Th1 differentiation, reduced IFNγ expression, and decreased protective capacity upon adoptive transfer. The authors argued that the primary mechanism responsible for these activities could be the modification of the signaling pathway downstream of PGE2 receptor 4 (EP4). However, further studies will be required to verify this assumption [40].

**Table 1:** Antimicrobial activity of NSAIDs.

Drug	Drug in Combination	Kind of Study	Bacterial Inhibition	Strains Tested	Refs.
**Acetylsalicylic acid**	-	In vitro	XTT reduction (%) by biofilms after 48 h ranging from 29.02 to 54.12	*C. albicans* (ATCC 10231, ATCC 10231, ATCC 90028, ATCC 24433, 17a18), *C. glabrata* (ATCC 15126, 18a10) *C. guilliermondii* (ATCC 6260, a83, a410)	[35]
**Acetylsalicylic acid**	**Anidulafungin**	In vitro	XTT reduction (%) by biofilms after 48 h ranging from 41.54 to 82.81	*C. albicans* (ATCC 10231, ATCC 10231, ATCC 90028, ATCC 24433, 17a18), *C. glabrata* (ATCC 15126, 18a10) *C. guilliermondii* (ATCC 6260, a83, a410)	[35]
**Diclofenac**	-	In vitro	XTT reduction (%) by biofilms after 48 h ranging from 44.54 to 60.98	*C. albicans* (ATCC 10231, ATCC 10231, ATCC 90028, ATCC 24433, 17a18), *C. glabrata* (ATCC 15126, 18a10) *C. guilliermondii* (ATCC 6260, a83, a410)	[35]
**Diclofenac**	**Anidulafungin**	In vitro	XTT reduction (%) by biofilms after 48 h ranging from 54.28 to 71.04	*C. albicans* (ATCC 10231, ATCC 10231, ATCC 90028, ATCC 24433, 17a18), *C. glabrata* (ATCC 15126, 18a10) *C. guilliermondii* (ATCC 6260, a83, a410)	[35]
**Diclofenac**	**Anidulafungin**	In vitro	MIC values ranging from 1.02 μg/mL to 2.05 μg/mL	*C. albicans* (ATCC 10231, ATCC 90028, A18, 10A12, 810), *C. glabrata* ATCC 15126, *C. tropicalis* (ATCC 750, 810) *C. kefyr* ATCC 204093, *C. krusei* (ATCC 6258, 31A29), *C. parapsilosis* (11A13, 1A1, 911, 910)	[36]
**Diclofenac**	*M. piperita* Essential Oil	In vitro	MIC values ranging from 0.05 μg/mL to 0.51 μg/mL	*C. albicans* (ATCC 10231, ATCC 90028, A18, 10A12, 810), *C. glabrata* ATCC 15126, *C. tropicalis* (ATCC 750, 810) *C. kefyr* ATCC 204093, *C. krusei* (ATCC 6258, 31A29), *C. parapsilosis* (11A13, 1A1, 911, 910)	[36]
**Diclofenac**	*P. graveolens* Essential Oil	In vitro	MIC values ranging from 0.05 μg/mL to 0.41 μg/mL	*C. albicans* (ATCC 10231, ATCC 90028, A18, 10A12, 810), *C. glabrata* ATCC 15126, *C. tropicalis* (ATCC 750, 810) *C. kefyr* ATCC 204093, *C. krusei* (ATCC 6258, 31A29), *C. parapsilosis* (11A13, 1A1, 911, 910)	[36]
**Diclofenac**		In vitro	MIC values ranging from 0.05 μg/mL to 0.82 μg/mL	*C. albicans* (ATCC 10231, ATCC 90028, A18, 10A12, 810), *C. glabrata* ATCC 15126, *C. tropicalis* (ATCC 750, 810) *C. kefyr* ATCC 204093, *C. krusei* (ATCC 6258, 31A29), *C. parapsilosis* (11A13, 1A1, 911, 910)	[36]
1.25% **Diclofenac** solution		In vitro	Reduction biofilm percentage of colony-forming units amounting to 84.71%	*E. faecalis* ATCC 29212	[37]
2.5% **Diclofenac** solution		In vitro	Reduction percentage of colony-forming units amounting to 90.42%	*E. faecalis* ATCC 29212	[37]
5% **Diclofenac** solution		In vitro	Reduction percentage of colony-forming units amounting to 98.62%	*E. faecalis* ATCC 29212	[37]
**Ibuprofen**		In vitro	XTT reduction (%) by biofilms after 48 h ranging from 16.26 to 64.43	*P. aeruginosa* (PAO1, M5715, T63547, H25815), *B. cenocepacia* K562, *B. multivorans* SH2, *B. cepacia* 1753, *B. cenocepacia* HI4277	[38]
**Ibuprofen**		In vitro	XTT reduction (%) by biofilms after 48 h ranging from 51.31 to 64.22	*P. aeruginosa* (PAO1, M5715, T63547, H25815), *B. cenocepacia* K562, *B. multivorans* SH2, *B. cepacia* 1753, *B. cenocepacia* HI4277	[38]
**Ibuprofen**			Number of log CFU/mL after treatment with IBU at 100 µg/mL ranging from 1.08 E to 7.94 E	*P. aeruginosa* (PAO1, M5715, T63547, H25815), *B. cenocepacia* K562, *B. multivorans* SH2, *B. cepacia* 1753, *B. cenocepacia* HI4277	[38]
**Ibuprofen**		In vivo	Oral delivery of ibuprofen achieves therapeutic concentrations in serum (124.22 ± 15.40 µg/mL at 1 h post-treatment), reduces the bacterial burden (in lung and spleen), and improves survival in *P. aeruginosa* PAO1-infected mice	*P. aeruginosa* PAO1	[38]
**Ibuprofen** incorporated in epichlorohydrin-crosslinked chitosan microspheres		In vitro	Inhibition using 1 × 10^−4^ mol/L of microspheres	*E. coli*, *S. aureus*	[39]
**Celecoxib**		In vitro	Impairs immune memory and affects CD4 T-cell phenotype, reduces IFNγ expression, and decreases protective capacity upon adoptive transfer in treated mice	*M. tuberculosis* ATCC 35801	[40]

## 3. Antidepressants and Antipsychotics

Numerous evidence supports the theory that serotonin reuptake inhibitors, the main antidepressant class of drugs, are efficient antimicrobial drugs, attributable to their activity in the suppression of efflux pumps [41,42,43] (Table 2). In 2015, Ayaz et al. [44] explored the potential activity of sertraline against ATCC strains, clinical isolates of *S. aureus*, *E. coli,* and *P. aeruginosa*, alone and in combination with seven antibiotics. Moreover, they examined the potential antifungal activity against the mean diameter of inhibitory *Aspergillus niger*, *Aspergillus fumigatus*, *Aspergillus flavus*, and *Fusarium solani*. To determine the intrinsic antibacterial activity of sertraline against *S. aureus*, agar dilution and well assay methods were carried out. The diameter of inhibitory zones (DIZ) augmented with the increasing concentrations of sertraline. An interesting concentration-dependent activity of sertraline was also detected against *E. coli* 8739 and *P. aeruginosa*. For further development of the antimicrobial activity of sertraline, they examined 28 bacterial strains, including 3 ATCC and 25 clinical isolates, and 13 fungal strains. The MICs values detected for sertraline against *S. aureus* ATCC 6538, *E. coli* ATCC 8739, and *P. aeruginosa* ATCC 9027 were 20, 40, and 60 μg/mL, respectively. Concerning the clinical isolates as *S. aureus*, the inhibition ranged from 22% to 55.5%. Sertraline achieved an inhibition rate of 50% against clinical isolates of *E. coli* at 60 μg/mL. Regarding fungal strains, Minimum Fungicidal Concentrations (MFCs) were 20, 40, and 80 μg/mL for *A. niger*, 80 μg/mL and 100 μg/mL for *A. fumigatus*, 60 and 80 μg/mL for *A. flavus*, and 80 μg/mL for *F. solani*. Dunnett’s multiple comparison test performed for the comparison of positive control with the test groups, underlining a substantial increase in the susceptibility pattern of *S. aureus* 6538. Furthermore, the authors evaluated the synergistic effect of sertraline in combination with well-known antimicrobial agents. Indeed, DIZ for ciprofloxacin alone amounted to 21.50 ± 0.70 mm, which was raised with the addition of sertraline in a concentration-dependent manner. Furthermore, they added 60 μg/mL of sertraline, noticing a significant increase in the inhibitory zone (30 ± 4.24 mm). Similarly, levofloxacin, norfloxacin, and moxifloxacin showed a noteworthy synergy when combined with sertraline at a concentration of sertraline of 80 μg/mL. Moreover, gentamicin activity improved due to the presence of sertraline at 40 μg/mL. A similar trend was followed for the effect against *E. coli* 8739. The DIZ for ciprofloxacin in the absence of sertraline amounted to 20 ± 1.41 mm, rising to 23 ± 1.41 mm with the addition of 20 μg/mL of sertraline. Levofloxacin, norfloxacin, and gentamicin also demonstrated an effective synergy when combined with sertraline, respectively. The antimicrobial activity of moxifloxacin against *E. coli* 8739 achieved an inhibitory zone of 32 ± 1.41 mm when combined with 80 μg/mL of sertraline. Good results were also obtained for *P. aeruginosa* 9027. DIZ for levofloxacin and moxifloxacin rose with the addition of sertraline 40 μg/mL, SR 20 μg/mL, and sertraline 60 μg/mL of sertraline, respectively. In 2018, Hadera et al. [45] explored the potential antimicrobial activity of selected non-antibiotic drugs, among them, fluoxetine used alone and in combination with three conventional antimicrobial drugs, i.e., ciprofloxacin, benzyl penicillin, and fluconazole, against *E. coli*, *S. aureus*, and *C. albicans* strains. Fluoxetine possessed inhibitory effects against all the tested organisms. The association between fluconazole and fluoxetine against *C. albicans* seemed particularly interesting. By contrast, the association of benzyl penicillin + fluoxetine against *E. coli* was not effective. An increase in antimicrobial effectiveness was detected for penicillin + fluoxetine. In the same way, the association between fluconazole, propranolol, and fluoxetine led to a percentage increase in surface area of the zone of inhibition. Concerning the MICs values of fluoxetine, propranolol, and fluconazole non-antibiotics against *C. albicans*, the MIC of fluconazole alone was found to be 12.5 μg/mL, whereas in association with propranolol and fluoxetine, the MIC value of fluconazole decreased to 1.5625 μg/mL and 0.78125 μg/mL, respectively. The association between fluoxetine and fluconazole proved to be successful against *C. albicans*.

In 2018, de Sousa et al. [46] explored the potential antimicrobial activity and the antibiotic modulating effect of fluoxetine against standard and multi-resistant bacterial strains. The authors focused their efforts on finding a potential effect against *P. aeruginosa*, *S. aureus*, and *E. coli*. Furthermore, the estimation of fluoxetine modulatory activity was carried out by associating this drug with erythromycin, gentamicin, imipenem, norfloxacin, and tetracycline at sub-inhibitory concentrations. Data suggested that when fluoxetine was combined with gentamicin and erythromycin against *P. aeruginosa* and *E. coli*, synergistic effects were achieved, underlining that fluoxetine can regulate the effect of clinically used antibiotics. Concerning the mechanism of action, the authors argue that inhibition of efflux pumps is the method of action known and envisaged from studies in human cells. In fact, this mechanism may be responsible for synergy with antibiotics such as tetracyclines and fluoroquinolones. Furthermore, because SSRIs affect a number of processes engaged in product biosynthesis in microorganisms, it has been suggested that they act on basic metabolic processes, whether or not they are related to the absorption of substances.

In 2019, Gowri et al. [47] surveyed the possibility of sertraline counteracting fungal infections, such as the one caused by a multidrug-resistant fungal pathogen, *Candida auris*, and its ability to impede biofilm formation. Sertraline potency was examined against three diverse isolates of *C. auris*. Data evidenced a powerful antifungal effect of sertraline (MIC value of 20 μg/mL against *C. auris* 70 and 40 μg/mL against *C. auris* 33 and *C. auris* IL, respectively). Sertraline treatment suppressed *C. auris* yeast to hyphae conversion. Moreover, it was able to hamper 71% of biofilm formation. Another interesting outcome concerns the *C. auris* cell damage assessed with scanning electron microscope (SEM), whereas cell membrane damage was observed through flow cytometry with propidium iodide (PI) uptake assay. Moreover, other tests aimed at deeply exploring the mechanism of action, such as the sorbitol protection assay and ergosterol effect assay, indicated the inability of sertraline to impact the cell wall and not to bind to membrane ergosterol. However, docking studies demonstrated that sertraline could bind to the sterol 14 alpha demethylase, which takes part in ergosterol biosynthesis. Indeed, ergosterol, measured in treated cells, decreased by 5-5-fold.

Other studies on sertraline regarded its potentiality against *Helicobacter pylori*, a spiral bacterium causing gastric pathologies and extensively discussed in the literature due to its problem of antibiotic resistance [48]. The disk-diffusion method established that the obtained growth inhibition zones were directly proportional to the concentration used against *Helicobacter pylori* strains. In particular, the strain most susceptible to sertraline was *H. pylori* 7556 (CLR-resistant) with a MIC value of 2 µg/mL and a minimum bactericidal concentration (MBC) of 4 µg/mL. On the contrary, *H. pylori* 7471 (antibiotic-sensitive) achieved MIC and MBC values equal to 8 µg/mL, proving to be the less sensitive. Through a time-killing assay, it was possible to demonstrate that sertraline possessed a time-dependent and concentration-dependent bactericidal effect. Using a scanning microscope, it was possible to detect the capability of sertraline to change the morphology of both strains of *H. pylori* by decreasing the number of spirals in a concentration-dependent manner. Furthermore, in bacteria treated with sertraline MBCs (but not sub-MICs and MICs, MBCs), a morphological transition into coccoid forms occurred. Moreover, the analysis performed through the checkerboard assay estimated a synergistic/additive interaction between sertraline and four antibiotics, such as amoxicillin, clarithromycin, tetracycline, and metronidazole. According to the authors, this synergistic effect may be due to the ability of sertraline to inhibit protein translation as well as its capacity to interfere with efflux pumps.

A recent study focused on the antimicrobial activity of fluoxetine and paroxetine in association with ciprofloxacin [49]. The authors calculated MIC, MBC, fractional inhibitory concentration index (FICI), and tolerance level toward 11 bacterial standard reference strains from the American Type Culture Collection (ATCC) and five clinical isolates from patients admitted to a university hospital, two of them being multidrug resistant (MDR). Data suggested that fluoxetine and paroxetine presented antibacterial activity against both the standard ATCC Gram-positive and Gram-negative strains. Interestingly, MIC values were identified against *S. epidermidis* ATCC 12228 (MIC 64 µg/mL), *E. faecalis* ATCC 51299 (MIC 128 µg/mL), and *S. aureus* ATCC 25923 (MIC 64 and 128 µg/mL) when they were combined with the ciprofloxacin or sulfamethoxazole + trimethoprim antibiotic, underlining the occurrence of a synergistic effect of the combination of a non-antibiotic with an antibiotic drug. In a study by Machado et al. in 2020 [50], amitriptyline, a tricyclic antidepressant, proved to be effective against both Gram-positive and Gram-negative strains. Specifically, the highest antibacterial activity transpired against the carbapenem-producer clinical isolates of *Klebsiella pneumoniae*. Indeed, the association of amitriptyline with colistin, considered a “last-resource” antibiotic in the treatment of acute infections caused by MDR Gram-negative microorganisms, exhibited MIC amounting to 4 µg/mL against two isolates, *Klebsiella pneumoniae-8* and *Klebsiella pneumoniae*.

De Andrade Neto et al. studied the possible mechanism of action of fluoxetine against MRSA. In fact, they carried out cytometric analysis revealing that treatment with fluoxetine resulted in alterations in the integrity of plasma membranes and DNA damage, which provoked cell death, possibly by apoptosis [51].

Phenothiazines are compounds mainly used to treat psychotic disorders, and their primary antipsychotic action seems to be based on the suppression of dopamine by blocking the dopaminergic receptors. Some studies demonstrated that many derivatives, such as chlorpromazine, thioridazine, and trifluoperazine, also possessed antimicrobial activities [15,25,41]. In terms of the mechanism of action, phenothiazines are able to reduce pathogen adhesion in endothelial cells. In addition, they interfere with the activity of calcium-dependent ATPase, resulting in the acidification of phagolysosomes, the activation of hydrolases, and finally, leading to inhibition of the replication of bacterium. Moreover, they can also block efflux pumps, which take into account antibiotic resistance of bacteria [16]. In 2020, Ruth et al. investigated the efficacy of thioridazine, which has previously shown beneficial effects against *M. tubercolosis* [52,53] toward *Mycobacterium avium* through in vitro and ex vivo studies. Thioridazine has been tested alone and in association with some established antimycobacterial drugs, such as clarithromycin or rifampin. FICI values were found to be less than 0.5, confirming the synergistic action. The MIC of thioridazine against *M. avium* ATCC 700898 was 16 µg/mL, and the MBC was 32 µg/mL, defining it as bactericidal. Ex vivo studies outlined that the combination of thioridazine and clarithromycin lowered the bacterial burden more than either compound alone. Moreover, the research group demonstrated that thioridazine was able to hamper ethidium bromide efflux, demonstrating, therefore, that the main mechanism of action consists of the inhibition of the efflux pumps. The authors stated that this mode of action could be a successful means of improving the effectiveness of existing multi-drug treatment [54]. In the same year, Nistorescu et al. [55] analyzed the quantitative antimicrobial activity of chlorpromazine through the laser irradiation technique (widely employed in recent years to lead to photodegradation of the parental compounds into photoproducts with possible antimicrobial properties) [56,57,58]. In addition, they evaluated the effect of both solutions impregnated on a cotton patch, cannula, and urinary catheter (due to the existence of a large number of urinary tract infections) against Gram-positive *S. aureus* and Gram-negative *P. aeruginosa* and *E. coli*. Chlorpromazine antimicrobial action was ameliorated by laser exposure in all the experiments. For instance, concerning *E. faecalis* strains, the MIC value of the irradiated solution went from 50 µg/mL (non-irradiated) to 6.25 µg/mL, and a similar trend of results was obtained for the other strains. Furthermore, docking studies demonstrated that the enhanced inhibitory action of the irradiated compound was a result of the overall effect of the photoproducts on the biological target. The capability of chlorpromazine to impede the biofilm formation on urinary catheter was also demonstrated by Sidrim et al. [59] in a previous work, in which this drug significantly suppressed the growth of *E. coli*, *Proteus mirabilis*, and *Klebsiella.* Tozar et al. in 2020 examined the anti-staphylococcal activity and mode of action of thioridazine photoproducts after different periods of laser irradiation. Water solutions of the drug were explored for their antimicrobial and efflux inhibitory activity against a panel of bacteria of clinical relevance. Their findings highlighted an improved antibacterial effect of the thioridazine photoproducts against Gram-positive bacteria. Surprisingly, this activity was higher than ciprofloxacin for methicillin- and ciprofloxacin-resistant *S. aureus*. Docking studies underlined the inhibition of Penicillin-binding proteins PBP3 and PBP2a by sulforidazine as a possible mode of action against *S. aureus* and MRSA strains, respectively [60].

**Table 2:** Antimicrobial activity of antidepressants and antipsychotic drugs.

Drug	Drug in Combination	Kind of Study	Bacterial Inhibition	Strains Tested	Refs.
**Sertraline**		In vitro	Diameter of inhibitory zones up to 26 mm 4–20 μg/mL	*S. aureus* ATCC 6538, *E. coli* ATCC 8739, *P. aeruginosa* ATCC 9027 *A. niger*, *A. fumigatus*, *A. flavus*, *F. solani C. auris* 70, *H. pylori*	[44,47,48]
**Fluoxetine**		In vitro	Diameter of inhibitory zones 12–34 mm	*S. aureus* ATCC 25923, *E. coli* ATCC 25922, *C. albicans* ATCC 10231	[45]
**Fluoxetine**	**Fluconazole**	In vitro	0.78125 μg/mL	*S. aureus* ATCC 25923, *E. coli* ATCC 25922, *C. albicans* ATCC 10231	[45]
**Fluoxetine**		In vitro	102–256 μg/mL	*E. coli* ATCC 25922, *S. aureus* ATCC 6538, *P. aeruginosa* ATCC 25923 and multi-resistant strains *E. coli* 06, *S. aureus* 10, *P. aeruginosa* 24	[46]
**Fluoxetine**		In vitro	32–512 μg/mL	*E. coli* (ATCC 35218, ATCC 25922), *K. pneumoniae* ATCC 700603, *P. aeruginosa* ATCC 27853, *S. aureus* (ATCC 25923, ATCC 29213), *E. faecalis* (ATCC 29212, ATCC 51288), *S. epidermidis* ATCC 12228, *M. luteus* ATCC 7468 *and B. cereus* ATCC 14579, 5 MDR clinical isolates	[49]
**Paroxetine**		In vitro	32–512 μg/mL	*E. coli* (ATCC 35218, ATCC 25922), *K. pneumoniae* ATCC 700603, *P. aeruginosa* ATCC 27853, *S. aureus* (ATCC 25923, ATCC 29213), *E. faecalis* (ATCC 29212, ATCC 51288), *S. epidermidis* ATCC 12228, *M. luteus* ATCC 7468 *and B. cereus* ATCC 14579, 5 MDR clinical isolates	[49]
**Amitriptyline**		In vitro	32–512 μg/mL	11 ATCC standard strains, 15 clinical isolates of KPC, 25 of SCoN	[50]
**Amitriptyline**	**Ciprofloxacin**	In vitro	64–256 μg/mL	11 ATCC standard strains, 15 clinical isolates of KPC, 25 of SCoN	[50]
**Amitriptyline**	**Sulfametoxazole + trimethoprim**	In vitro	64–512 μg/mL	11 ATCC standard strains, 15 clinical isolates of KPC, 25 of SCoN	[50]
**Thioridazine**		In vitro	1–16 μg/mL	3 ATCC rapidly growing mycobacteria *(M. abscessus CIP* 104536, *M. fortuitum* ATCC 6841, *M. peregrinum* ATCC 700686, *M. avium* (ATCC 70089816, B1610670, 74B16107, 2282B161, 0557732, B1701907), *M. chimaera* (DSM 4462316, B160155388, B160184894, B17072535), *M. intracellulare* (DSM 432234, B161255242, B1611688316, B16029695), *M. simiae* ATCC 252211, *M. xenopi* ATCC 192504	[54]
**Thioridazine-irradiated solution**		In vitro	0.25–50 μg/mL	*S. aureus* (ATCC 25923, ATCC 25923_EtBr), *S. epidermidis* (ATCC 12228, ATCC 12228_EtBr, SM1) *E. faecalis* ATCC 29212, *E. coli* ATCC 25922, *Salmonella enterica serotype Enteritidis* NCTC 13349, *Klebsiella aerogenes* ATCC 15038	[58]
**Chlorpromazine-irradiated solution**		In vitro	6.25–100 μg/mL	*S. aureus* ATCC 6538, MRSA1, MRSA2, *E. faecalis* ATCC 29212, *P. aeruginosa* ATCC 27853, *P. aeruginosa clinic1*, *P. aeruginosa clinic2*, *E. coli* ATCC 8739, *C. parapsilosis* ATCC 22019	[55]

## 4. Statins as Antimicrobial Agents

Statins, a class of lipid-lowering agents, which reduce heart-associated morbidity and mortality, have demonstrated, over the years, to possess anti-inflammatory and immunomodulatory activities. Furthermore, many researchers suggested a potential protective effect of statins against several infectious diseases [61] (Table 3). Among the disparate statins, atorvastatin and simvastatin in particular have been examined for their antimicrobial effects. Most of these studies estimated the potentiality of these compounds against Methicillin-sensitive *S. aureus* (MSSA), Methicillin-resistant *S. aureus* (MRSA), Vancomycin-susceptible *Enterococci* (VSE), Vancomycin-resistant *Enterococcus* (VRE), *Acinetobacter baumannii*, *S. epidermidis*, and *Enterobacter aerogenes* [62,63,64].

In 2018, Ko et al. [65] corroborated the antibacterial potentiality of statins by testing seven of them (atorvastatin, fluvastatin, lovastatin, pitavastatin, pravastatin, rosuvastatin, and simvastatin), along with three selected statin metabolites (lovastatin hydroxy acid sodium, pitavastatin lactone, and simvastatin hydroxy acid sodium SMV-OH acid) against some strains provoking skin and soft tissue infections (*S. aureus*, *E. coli*, *P. aeruginosa*, and *Serratia marcescens*). The best effect was accomplished with simvastatin and pitavastatin against *S. aureus*, with MICs amounting to 64 and 128 μg/mL, respectively. Furthermore, simvastatin hydroxy acid could reach an effect against *S. aureus*, *E. coli*, and *S. marcescens* at drug concentrations >256 μg/mL. Concerning the structure–activity relationship analysis, the research group hypothesized that statins’ antibacterial action may involve disrupting the teichoic acid structures or reducing the number of alanine residues on Gram-positive bacterial cell surfaces, resulting in biofilm formation decrease.

In 2020, Akbarzadeh et al. [66] prepared and optimized a niosomal formulation (a particular kind of drug delivery system employed for the topical delivery of lipophilic drugs) of simvastatin. Their findings suggested that niosomes notably reduced the drug-releasing rate and improved the antimicrobial effect against *S. aureus* and *E. coli* (MIC values amounting to 31.12 and 7.78, respectively). Furthermore, they highlighted that the release pattern of drug followed the Higuchi kinetic model, suggesting that drug release occurred through diffusion. Another study proved that simvastatin could fight infectious diseases generated by *E. faecalis* strains when combined with Ag^+^. This association was made possible by poly (lactide-co-glycolide) (PLGA) submicron particles carrying both Ag^+^ and simvastatin (AgS-PLGA). The release of the treatment could last for 24 h, also improving the antibacterial properties. In addition, AgS-PLGA demonstrated no cytotoxicity on MC3T3-E1 cells and a slight suppressive effect on RAW-264.7 cells and could decrease the secretion of IL-6 and IL-1b of RAW-264.7 cells [67]. Similarly, Figueiredo et al. [68] analyzed the antimicrobial effects of silver nanoparticles synthesized with Fusarium oxysporum (AgNPbio) in association with simvastatin against reference and multidrug-resistant bacterial strains. Results indicated that simvastatin possessed MICs ranging from 0.062 to 0.25 mg/mL against Methicillin-resistant *S. aureus* (MRSA). AgNPbio with a size of 77.68 ± 33.95 nm and zeta potential −34.6 ± 12.7 mV displayed a MIC of 0.212 mg/mL against *S. aureus*, including MRSA strains. Simvastatin-AgNPbio revealed a synergistic effect, with a notable antibacterial activity against *E. coli*, producing extended-spectrum beta-lactamase (ESBL). Moreover, through the scanning electron microscopy, it was possible to see the formation of cell surface protrusions, numerous lysed cells, and cell debris after treatment with AgNP-Bio and the formation of a large amorphous mass after treatment with simvastatin in MRSA, suggesting a possible interference with permeability of the bacterial cell membrane.

Another winning strategy turned out to be the association between statins and triazenes (TZC). In particular, a new TZC complex {[1-(4-bromophenyl)-3-phenyltriazene N3-oxide-κ2 N1,O4](dimethylbenzylamine-κ2 C1,N4)palladium(II)} (Pd(DMBA)LBr), when combined with simvastatin against several ATCC strains, exhibited a FICI value of <0.5, and MIC amounted to 16 µg/mL in six samples [69]. In 2019, atorvastatin was used in association with conventional antimicrobial treatments against *Helicobacter pylori* in a randomized controlled clinical trial. The study was performed on a total of 220 patients with *H. pylori* infection, among whom 110 in the control group received a 14-day regimen of amoxicillin, clarithromycin, bismuth, and esomeprazole, and 110 patients in the intervention group received 40 mg of atorvastatin daily plus the antibiotic regimen for 14 weeks. After a month of treatment, *Helicobacter pylori* eradication rate in the intervention and control groups was about 78.18% and 65.45%, respectively, with a significant difference in terms of non-ulcer dyspepsia between the groups, without discrepancies concerning age, gender, and body mass index between the two groups. The authors stated that the potential mechanism of action involved in statins’ antimicrobial effects could be attributable to their immunomodulatory activities [70].

**Table 3:** Statins with antimicrobial activity.

Drug	Drug in Combination	Kind of Study	Bacterial Inhibition	Strains Tested	Refs.
**Atorvastatin**		In vitro	15.62–229.17 μg/mL	*E. coli* ATTC 35218, *P. aeruginosa* ATTC 9027, MSSA ATTC 25213, MRSA ATTC 43300, *S. pneumoniae* ATTC 25923, VSE ATTC 19433, VRE ATTC 51299, *A. baumannii* ATTC, 17978, *K. pneumoniae* ATTC 13883, 80 clinical isolates	[64]
**Atorvastatin**	**amoxicillin, clarithromycin, bismuth, and esomeprazole**	In vivo	eradication rate: 65.45–78.18%	Patients with *H. pylori* infection	[70]
**Simvastatin**		In vitro	26.04–291.67 μg/mL	Patients with *H. pylori* infection	[70]
**Simvastatin**		In vitro	15.65–31.25 μg/mL	5 ATCC standard strains of *S. aureus* and 5 clinical isolates of sputum and blood, culture	[62]
**Simvastatin**		In vitro	64 μg/mL	*S. aureus* ATCC 29213	[65]
**Simvastatin niosomal formulation**		In vitro	7.78–31.12 μg/mL	*S. aureus* ATCC 6538 and *E. coli* ATCC 25922	[66]
**Simvastatin poly** (**lactide-co-glycolide**) (**PLGA**) **submicron particles with Ag^+^**		In vitro	100–150 μg/mL	*E. faecalis* ATCC 29212	[67]
**Simvastatin silver nanoparticles synthesized with Fusarium oxysporum** (**AgNP-Bio**)		In vitro	0.062 to 0.25 μg/mL	*S. aureus* MSSA (ATCC 25923, ATCC 29213), *E. coli* ATCC 25922, and extended-spectrum beta-lactamases *E. coli-producing* (ESBL 176)	[68]
**Simvastatin**	**TZC complex {[1-(4-bromophenyl)-3-phenyltriazene N3- oxide-κ2 N1,O4](dimethylbenzylamine-κ2 C1,N4)palladium(II)} (Pd(DMBA)LBr)**	In vitro	16–512 μg/mL	*Bacillus cereus* ATCC 14579, *Enterobacter hormaechei* ATCC 700323, *Enterococcus casseliflavus* ATCC 700327, *E. faecalis* (ATCC 29212, ATCC 51299), *E. coli* (ATCC 25922, ATCC 35218), *Klebsiella pneumoniae* ATCC 700603, *Micrococcus luteus* ATCC 7468, *P. aeruginosa* ATCC 27853, *Salmonella typhimurium* ATCC *14028*, *Salmonella* spp. ATCC 52117, *S. aureus* (ATCC 25923, ATCC 29213. BAA 1026, BAA 976, BAA 977), *S. epidermidis* ATCC 12228 and against 10 coagulase-negative staphylococci isolates in new-born blood cultures in 2014	[69]
**Rosuvastatin**		In vitro	104.17–500 μg/mL	*E. coli* ATTC 35218, *P. aeruginosa* ATTC 9027, MSSA ATTC 25213, MRSA ATTC 43300, *S. pneumoniae* ATTC 25923, VSE ATTC 19433, VRE ATTC 51299, *A. baumannii* ATTC 17978, *K. pneumoniae* ATTC 13883, 80 clinical isolates	[64]
**Pitavastatin**		//	128 μg/mL	*S. aureus* ATCC 29213	[65]

## 5. Other Compounds

Starting from the assumption that *Streptococcus mutans* represents the main cause of dental caries and plays a key role in the multispecies biofilm (known as dental plaque), Saputo et al. [71] performed the first high-throughput drug screening on *S. mutans* by selecting 853 FDA-approved drugs and using an adenylate-kinase-based assay to detect cell lysis when exposed to the Selleck library (Selleck Chemical, Houston, TX, USA). Results suggested that *S. mutans* was susceptible to 126 drugs, classified into six categories: antibacterials, antineoplastics, ion channel effectors, other antimicrobials, antifungals, and others. The research group performed other tests to detect a possible activity against *S. mutans* biofilm cultures. Among all the tested compounds, 24 hampered biofilm formation, 6 killed pre-existing biofilms, 84 showed biofilm inhibition and killing activity, and 12 possessed no effects against biofilms. Concerning the class of the ion channel effectors, they found that the addition of felodipine, a calcium channel blocker, to planktonic cultures of *S. mutans* prevented the growth at a concentration of 32 µg/mL, while concentrations at 0.5 × MIC reduced biofilm formation. They also studied the possible use of zinc pyrithione, an antiseborrheic employed in topical formulations, endowed with bacteriostatic properties against streptococci and staphylococci due to the increase in membrane permeability in zinc-pyrithione-treated cells, the ability to mediate the influx of damaging metal ions into the cell, and the capacity to chelate metals and transport them across membranes. Results suggested that in *S. mutans*, zinc pyrithione suppressed growth at 1 µg/mL with a bactericidal effect at 2 µg/mL, while biofilm formation was hampered at concentrations as low as 0.5 µg/mL (MBIC).

Concerning the antineoplastic drugs, this research group detected for the first time the antimicrobial activity of ponatinib, which caused the inhibition of *S. mutans* planktonic cultures at a MIC value of 8 µg/mL and impeded biofilm formation (MBIC 4 µg/mL), as well as inducing adenylate kinase (AK) release from preformed biofilms. Furthermore, they went into the potential antimicrobial effect of 6-imidazole derivatives (butoconazole, clotrimazole, econazole, fentriconazole, miconazole, and ticonazole) against *S. mutans*. Their findings underlined that all these drugs exhibited low MICs against *S. mutans* (8 µg/mL or lower; the MIC of fentriconazole was 4 µg/mL). Moreover, all these drugs prevented *S. mutans* biofilm formation and promoted AK release because of exposure to preformed biofilms. Other interesting findings suggested that statins, such as lovastatin, simvastatin, and atorvastatin, possessed lytic activity against *S. mutans*, as revealed by the AK assay. Further investigations, however, outlined that statins, including simvastatin, suppressed growth inhibition less, but they considerably inhibited biofilms at concentrations below the MIC (as low as 25 µg/mL). At the same, simvastatin, at a concentration of 50 µg/mL, decreased biofilm formation by 90% relative to the control. Moreover, they explored the capability of disulfiram to release AK when exposed to planktonic cultures of *S. mutans*. They assessed whether disulfiram possessed a comparable mechanism in *S. mutans* by using copper in the standard fractional inhibition concentration (FIC) assay. When copper was absent, the MIC of disulfiram against planktonic cells amounted to 16 g/mL. The association between disulfiram and copper led to a color change in the growth medium, indicative of breakage of the disulfide bond and coordination of copper, as previously reported [72]. The combination of copper and disulfiram resulted in synergistic activity, with a FIC of 0.375. A similar result was obtained when disulfiram was used in conjunction with copper against biofilm cultures (FIC 0.313), as 0.625 mM (106.55 g/mL) copper and 2 g/mL disulfiram inhibited biofilm formation (MBIC).

In 2014, Cassetta et al. [73] outlined the possible repurposing of Auranofin (Table 4), a gold(I) complex in clinical use for the therapy of rheumatoid arthritis, as an antimicrobial agent. Chemically, Auranofin consists of a gold(I) center linearly coordinated with a triethylphosphine and a thiosugar ligand. Its advantageous pharmacological properties are due to the presence of the strong phosphine ligand, whereas the thiosugar ligand is a weaker ligand and may be released more easily. This release enables a coordination position for gold(I) binding to biomolecules [74]. The purpose of their work was to test the potential efficacy of Auranofin against a few representative bacterial strains, such as *E. coli* ATCC 25922, *S. aureus* ATCC 25923, *S. aureus* USA 300, *S. epidermidis* ATCC 12228, *S. epidermidis* ATCC 35984 (biofilm producer and five recent clinical isolates of methicillin-resistant *S. aureus* (MRSA)). To make a comparison, tests were also performed on AuClPEt3 (II), an auranofin analog where the thiosugar ligand is replaced by a chloride ligand. Results suggested that both compounds could not act against Enterobacteriaceae strains due to the high MIC values calculated for *E. coli* (8 mg/L). On the contrary, the compounds demonstrated a conspicuous activity against *S. aureus* ATCC 25923 strains, with MIC values amounting for Auranofin and for AuClPEt3. Further analysis carried out on the *Staphylococcus* genus, including S. epidermidis, MRSA, and recent clinical isolates of MRSA, demonstrated that both gold compounds were effective in inhibiting all tested strains, with MIC values in the range between 0.125 and 0.5 mg/L. Time–kill curve analysis suggested that the antimicrobial effect of both compounds was marked on *S. aureus* ATCC 25923 strains. Moreover, Auranofin hampered bacterial growth for 12 h at MIC (0.5 mg/L) and for 24 h at twice the MIC. Moreover, Auranofin showed a concentration-dependent bactericidal effect with sterilization at 6–12 h at concentrations >4 × MIC, with a reduction in the bacterial count of 3 log compared to the control in the first 6 h. The mechanism of action held responsible for auranofin’s potent activity is the disruption of thiol redox homeostasis in the host by the suppression of flavoenzyme thioredoxin glutathione reductase (TrxR) as described in *Schistosoma mansoni* [75,76].

Ethyl bromopyruvate (EBP) is a derivative of 3-bromopyruvic acid, an anti-cancer agent. It inhibits the Warburg effect, where cancer cells tend to promote glycolytic metabolism compared to more effective oxidative phosphorylation, which is generally preferred by non-tumoral cells [77]. Kumar et al. demonstrated its activity against pathogens, such as *E. faecium*, *S. aureus*, *K. pneumoniae*, *A. baumannii*, *P. aeruginosa*, and *Enterobacter* spp. And *M. tuberculosis*, with MIC values ranging from 32 to 64 mg/L. In particular, they proved a bactericidal effect of EBP against *S. aureus* and *M. tuberculosis*. They also assessed the in vivo efficacy of EBP in a neutropenic murine *S. aureus* thigh infection model, proving that after 24 h of treatment, EBP could achieve the same potency of vancomycin. Concerning the mechanism of action, they evidenced that EBP is able to target multiple enzymes taking part in cellular metabolism, among them, glycolytic and glyoxylate pathways, such as lactate dehydrogenase, succinate dehydrogenase, hexokinase, and glyceraldehyde-3-phosphate dehydrogenase (GAPDH), with GAPDH being the main target. Furthermore, they proved that EBP also targets iron uptake by reducing surface binding of transferrin, and ultimately, transferrin-associated iron acquisition in *M. tuberculosis*.

Niclosamide is a chlorinated salicylanilide endowed with anthelmintic and probable antineoplastic activity. Nowadays, it is used against most tapeworm infections, such as intestinal nematodes, filarial nematodes, flukes, and tapeworms. Niclosamide has been approved for nearly 50 years to treat these infections in humans [78]. Its best antimicrobial activity has been found against Gram-positive bacteria, such as clinical isolates of *S. aureus* MRSA MW2 (MIC amounting to 0.125 µg/mL) and *E. faecium* E007 (MIC amounting to 0.25 µg/mL) [79]. Another work studied the capability of niclosamide to counteract *H*. *pylori*. Niclosamide exhibited a MIC value of 0.25 µg/mL against *H*. *pylori* ATCC 49503. The main mechanism of action seems to be due to the disruption of H. pylori proton motive force. Tharmalingam et al., in their study, evaluated the in vivo efficacy of niclosamide in a *Galleria mellonella* model (larvae) of *H. pylori* infection. The niclosamide-treated group achieved a survival rate of 70% over a 5-day treatment [80]. In 2018, Ayerbe-Algaba et al. explored the synergistic effects of niclosamide and colistin against colistin-resistant strains (clinically isolated) of *A. baumannii* and *K. pneumoniae*. Interestingly, they found that niclosamide at 1–4 µM in combination with colistin enhanced the activity of colistin significantly. In these bacteria, niclosamide raised the proportion of negative charges on their cell walls and thus was able to potentiate the activity of colistin against colistin-resistant *A. baumannii* and *K. pneumoniae* [81]. The niclosamide mechanism of action involves the blockage of glucose uptake, thus acting as an uncoupling agent for energy-generating oxidative phosphorylation in intestinal worms, starving the worms of ATP [82].

Metformin is an approved drug for the treatment of type 2 diabetes mellitus (DM) and is known to be a reversible inhibitor of mitochondrial NADH dehydrogenase, resulting in lower ATP production [17]. Many studies tried to evaluate the potential role of metformin in patients with tuberculosis and DM. Indeed, it demonstrated a decrease in mortality and protective effects in DM patients following an anti-tuberculosis regimen [83,84,85]. Recently, He et al. explored the synergistic in vitro antimicrobial activity of triton X-100 and metformin against *E. faecalis* ATCC 29212 in normal and high-glucose conditions. They surmised that the antimicrobial activity of metformin against *E. faecalis* could be greatly enhanced by combining it with a very low concentration of TX-100, in both normal and high-glucose conditions (MIC values five times lower). Moreover, they demonstrated that the expression of some heat shock proteins, which are produced in response to *E. faecalis* infection, was markedly decreased. In fact, by downregulating the expression of stress genes and CcpA, the combination of TX-100 and metformin hampered the stress response and glycolytic capacity of *E. faecalis*, thereby decreasing their viability and proliferation and diminishing their pathogenicity and drug resistance. Furthermore, they ascertained the antibiofilm activity of this association against *E. faecalis* in a dentin biofilm model [86].

**Table 4:** Antimicrobial activity of other compounds.

Drug	Drug in Combination	Kind of Study	Bacterial Inhibition	Strains Tested	Refs.
**Auranofin**		In vitro	0.125–0.5 mg/L	*E. coli* ATCC 25922, *S. aureus* (ATCC 25923, USA 300), *S. epidermidis* (ATCC 12228, ATCC 35984 (biofilm producer)), 5 MRSA	[73]
**Niclosamide**	-	In vitro	0.125–64 µg/mL	*S. aureus* (MRSA MW2, Newman, RN4220, RN6390, USA100, USA300, USA400), *S. epidermidis* 9142, *E. faecium* E007, *K.* *pneumoniae* ATCC 77326, *A. baumannii* ATCC 17978, *P. aeruginosa* PA14, *E. aerogenes* EAE 2625 and 44 *S. aureus* clinical	[79]
**Niclosamide**		In vitro	0.25 µg/mL	*H. pylori* 60190 ATCC 49503	[80]
**Niclosamide 4** **µM**	**Colistin**	In vitro	MIC values ranging from <0.03 μg/mL to 0.125 μg/mL	Reference colistin-susceptible (Col-S) *A. baumannii* ATCC 17978 strain, and 13 clinical colistin-resistant (Col-R) A, reference Col-S *K. pneumoniae* CECT 997 strain, 1 Col-S and 2 Col-R clinical *K. pneumoniae* strains	[81]
**Metformin**	TX-100	In vitro	20 mg/mL	*E. faecalis* ATCC 29212	[86]

## 6. Conclusions

Drug repositioning is a versatile approach to identify new developmental paths for failed drug candidates or for compounds with a diverse therapeutic application. The return to already existing therapies has been growing in popularity over the last few years due to the need to decrease costs and therapeutic problems connected with antimicrobial resistance, one of the biggest pharmaceutical challenges of our times. For these reasons, the inspection of compounds approved for the therapy of other disorders is an attractive modality, which could guarantee prompt transference to the clinic. In this review, we explored the research regarding different classes of well-known drugs studied as potential effective antimicrobial agents. In particular, we focused our attention on some drugs belonging to NSAID, antidepressant, antipsychotic and statin classes, which exhibited great potentiality in fighting infectious diseases, including the antibiotic-resistant ones. Indeed, some of these studies have also been supported by clinical evidence. It is interesting that when associated with antibacterials, most of these drugs had their antimicrobial activity enhanced. The promising activity of some of these drugs on biofilm reveals their potential usefulness for biofilm prevention and control. Taking everything into account, the repositioning of already known drugs, due to its clear benefits, can be deemed an encouraging strategy against numerous infections and may improve the portfolio of pharmaceutical companies, decreasing the exigency for pharmacokinetic and toxicity studies, also solving sanitary problems of global concern. Nevertheless, it is a consensus in the global scientific community that this is only the starting point, and additional studies regarding mechanisms of action and in vivo studies, among others, are crucial for the safe use of these drugs.
